# Supplementary material for: A New Synuclein-Transgenic Mouse Model for Early Parkinson’s Reveals Molecular Features of Preclinical Disease
Source: Mol Neurobiol. 2020 Sep 30;58(2):576–602. doi: 10.1007/s12035-020-02085-z (PMC8219584; doi:10.1007/s12035-020-02085-z)
Supplement: Supplementary file 1 — (PDF 11.0 MB) [file 12035_2020_2085_MOESM1_ESM.pdf]

Supplementary figures and tables

August 1, 2020

Supplementary figures and tables

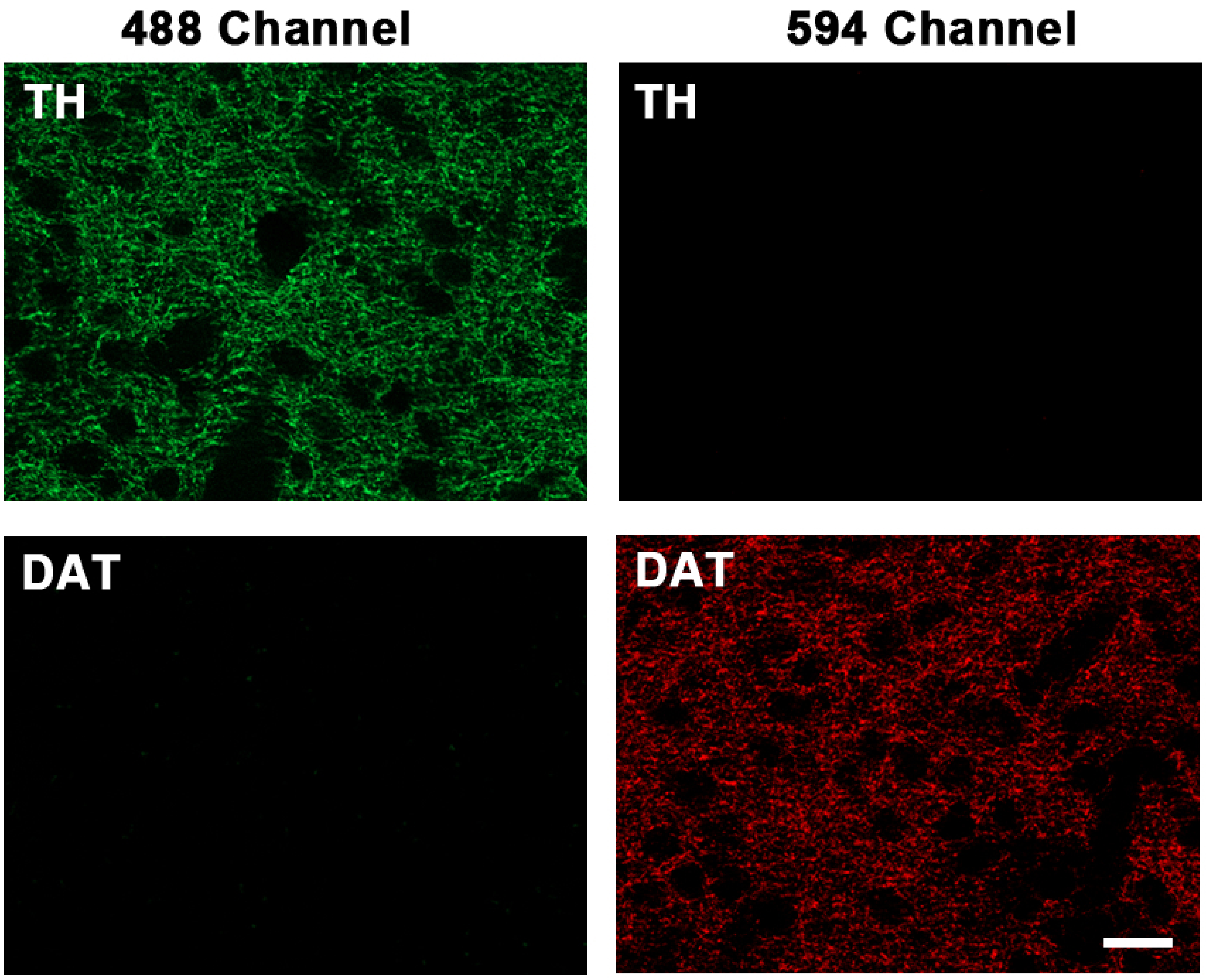

Figure S1: Fluorescent bleed-through control for striatal TH and DAT stainings. Sections at the level of the striatum were stained separately for TH, which was revealed by a secondary antibody coupled to a 488 nm emitting (green) fluorophore, or for DAT, which was revealed by a secondary antibody couple to a 594 nm emitting (red) fluorophore. Each staining was first imaged with their respective appropriate filter set, then with the filter set for the other fluorophore (green signals imaged with red filters, red signal imaged with green filter). The absence of signal when the stainings were imaged with inappropriate filter set confirms that there was no fluorescent bleed-through with the immunostaining protocol used.

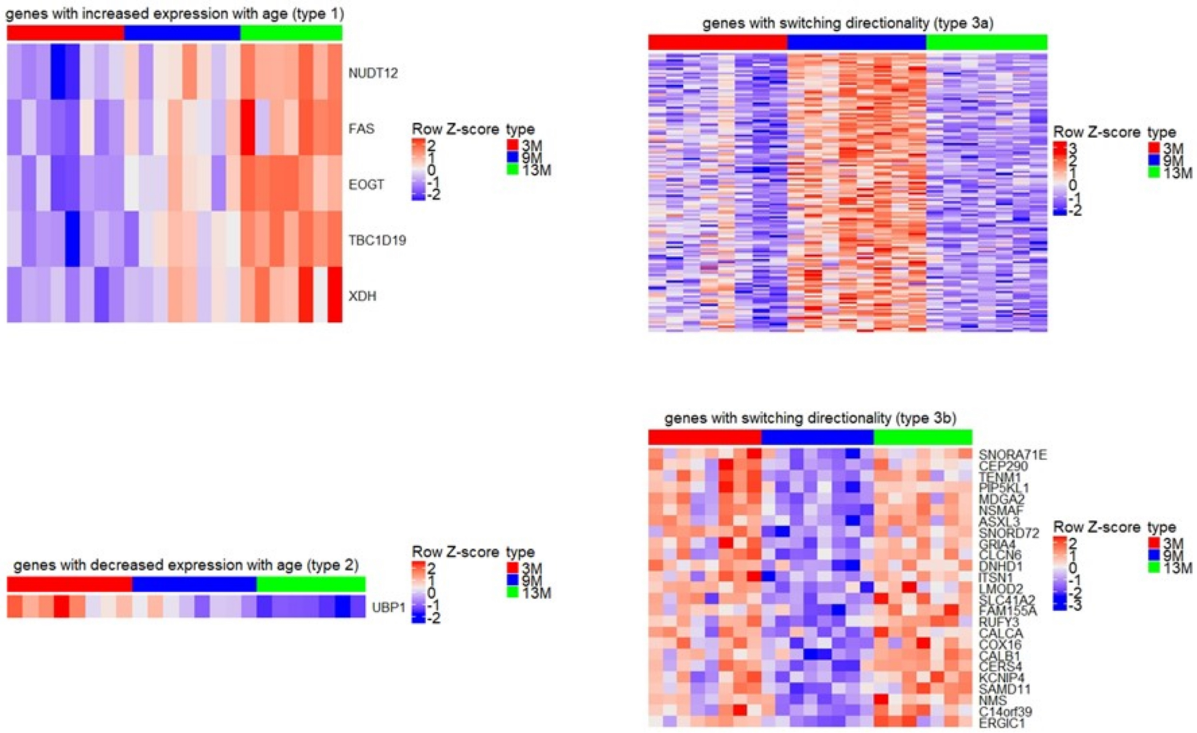

Figure S2: Heat maps (row z-scores) for: genes with increased expression with age (type 1, top left); genes with decreased expression with age (type 2, bottom left); genes with switching directionality up/down (type 3a, top right); genes with switching directionality down/up (type 3b, bottom right).

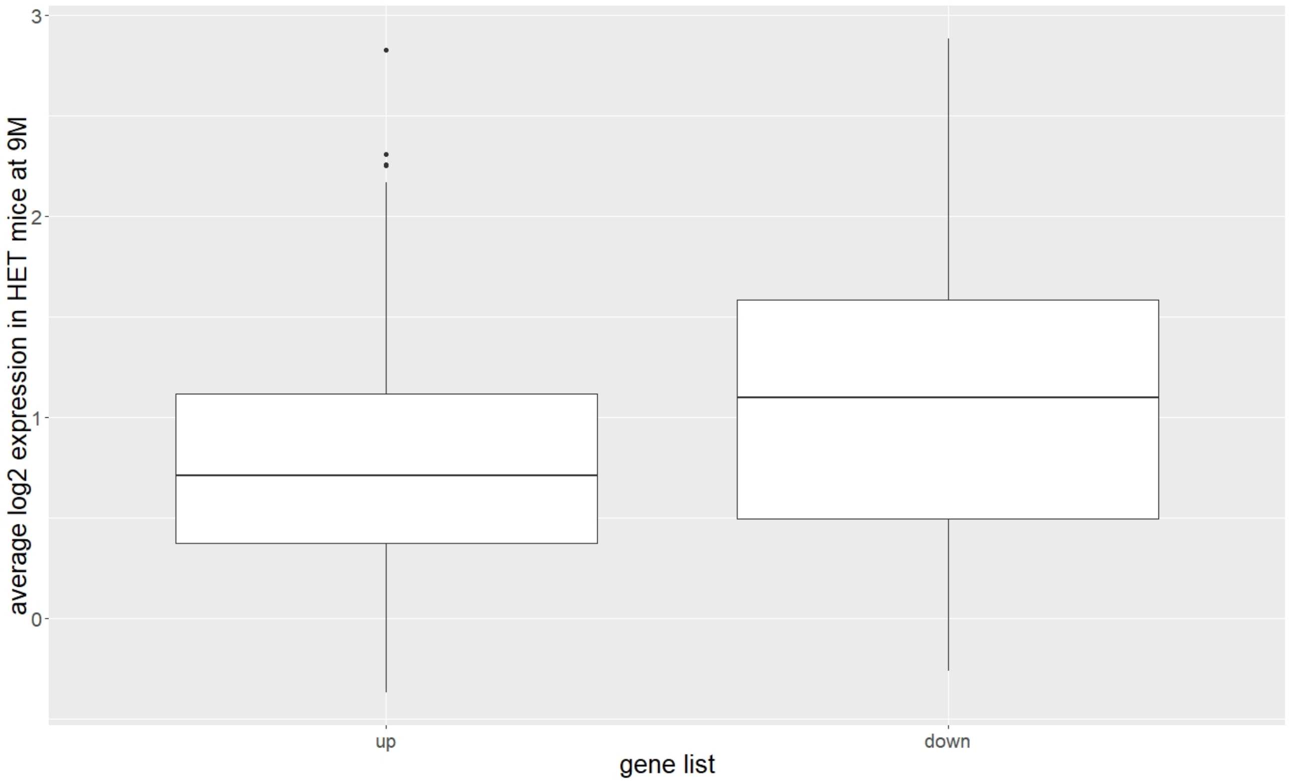

Figure S3: Boxplots of average log2 expression in HET mice at 9M. Left: genes with higher expression between 3M and 9M in HET only, but not differentially expressed between 9M and 13M; right: genes with lower expression between 3M and 9M in HET only, but not differentially expressed between 9M and 13M. The horizontal line in the middle of each boxplot represents the median (50% percentile) expression of the genes within the gene list; the box represents the interquartile range, i.e. the range between the 25% percentile and the 75% percentile; the whiskers represent the lowest and the highest value. Points represent outlying values.

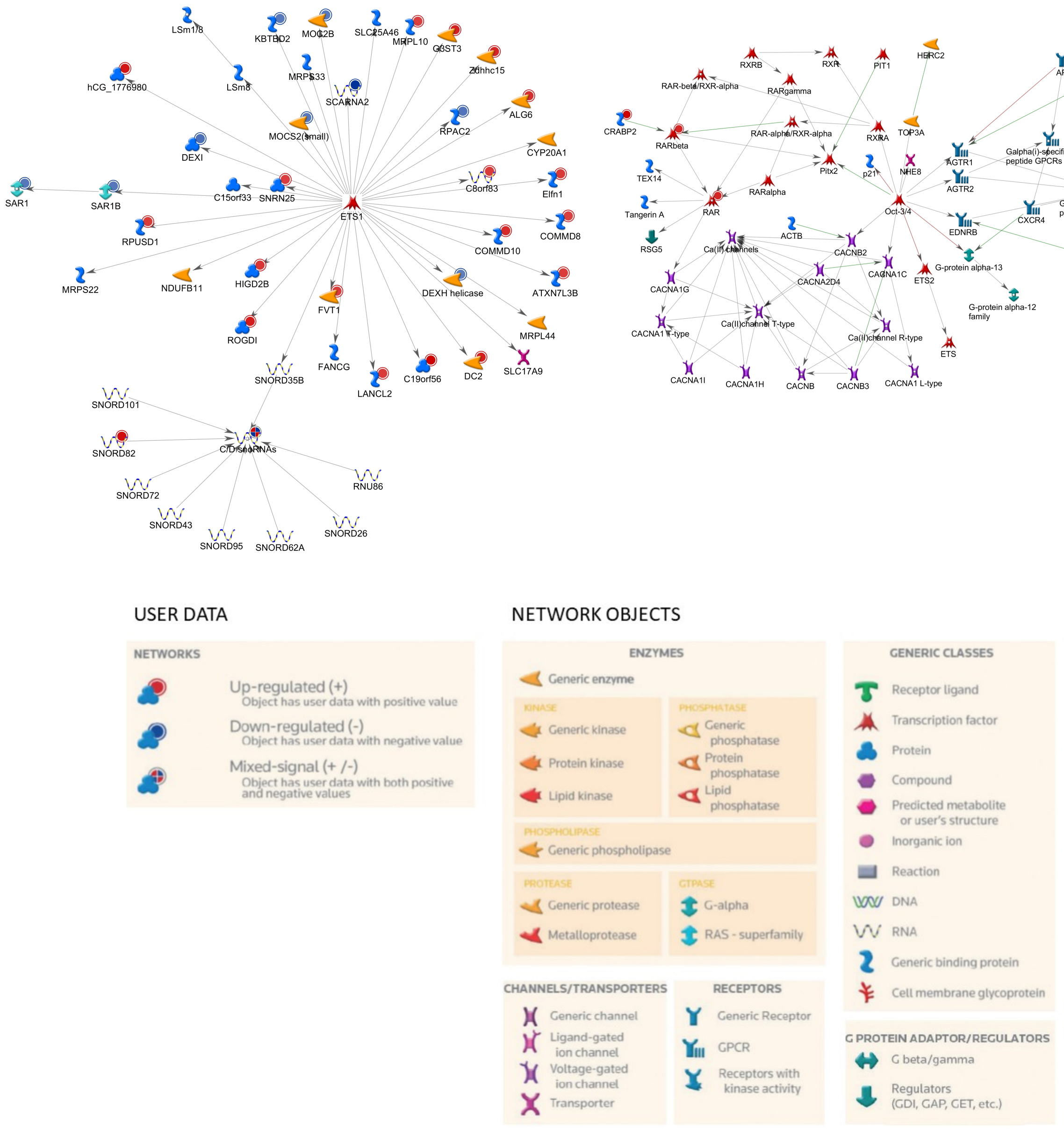

Figure S4: Network with the largest number of input gene products (gene products from DEGs) for 3M HET vs 3M WT, identified by GeneGO MetaCore^{TM}'s default "Analyze network" procedure. Gene products of DEGs are indicated with a circle. Left: 3M HET vs 3M WT; right: 9M HET vs 9M WT.

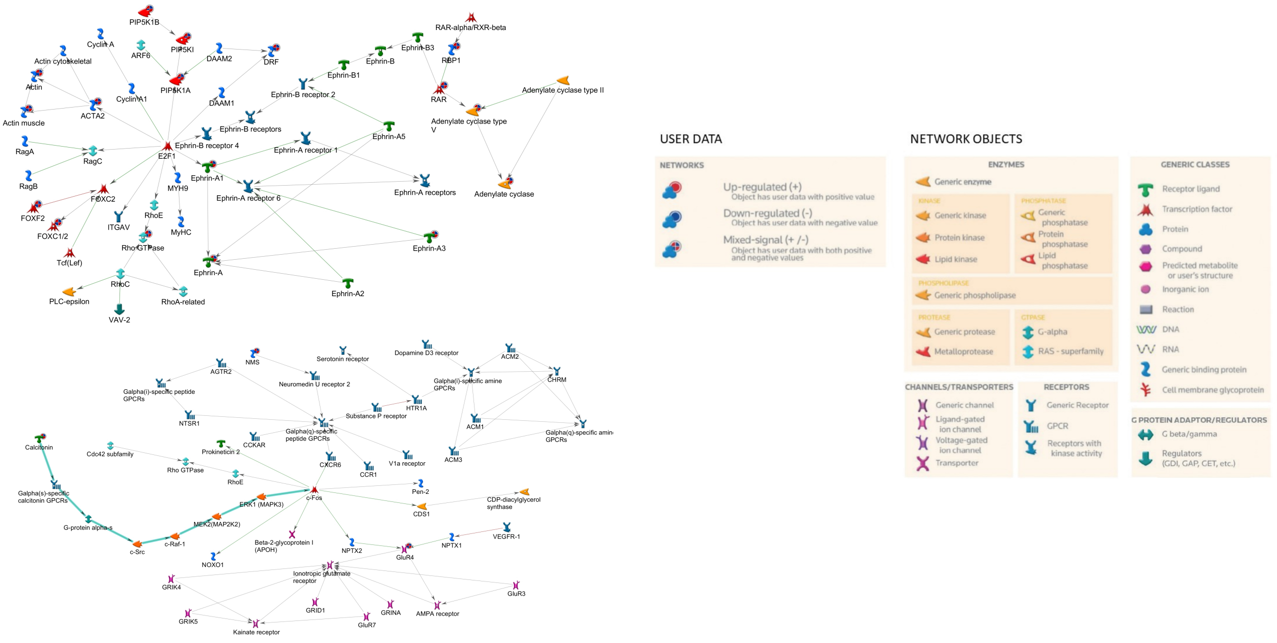

Figure S5: Network with the largest number of input gene products (DEGs) for age-dependent DEGs, identified by GeneGO MetaCore^{TM}'s default "Analyze network" procedure. Gene products of DEGs are indicated with a circle. Upper panel: type 3a; lower panel: type 3b. Red-blue: increased expression between 3 months and 9 months, decreased expression between 9 months and 13 months respectively. Cyan line: fragments of canonical pathways.

Table S1: Genes with human orthologues in Ensembl Biomart (`https://www.ensembl.org/biomart`), but classified as mouse specific in NCBI Homologene (`https://www.ncbi.nlm.nih.gov/homologene`). The fourth column indicates in how many databases used by HGNC (`https://www.genenames.org/`) the orthologues show up. The fifth column shows expression in brain in transcripts per million (TPM) according to the Human Proteome Atlas (`https://www.proteinatlas.org/ENSG00000069493-CLEC2D/tissue`).

mouse	human	DEG list(s)	HGNC	expression in brain (TPM)
Tmsb4x	TMSB4Y	9M HET vs 3M HET, 13M HET vs 9M HET, 9M WT vs 3M WT, 13M WT vs 9M WT	3	3
Rplp1	RPLP1	9M HET vs 3M HET, 9M WT vs 3M WT	11	430
Zfp947	ZNF34	13M HET vs 9M HET, 9M WT vs 3M WT	1	8.4
Josd1	JOSD1	13M HET vs 9M HET	11	39.5
Gm10230	SYCP3	9M WT vs 3M WT	3	—
Ptma	PTMA	9M WT vs 3M WT	7	624.3
Gm2030	SYCP3	9M WT vs 3M WT	3	—
Gm2042	PRAMEF8	9M WT vs 3M WT	2	—
Gm20738	SPIN2B	9M WT vs 3M WT	1	16.1
Vmn1r27	VN1R5	9M WT vs 3M WT	1	—
Zbed4	ZBED4	9M WT vs 3M WT	10	5.3
Clec2d	CLEC2D	13M WT vs 9M WT	6	2

Table S2: Mean and variance analysis of groups in the dataset. 2nd column: mean absolute logFC (average over all measured genes); 3rd column: mean absolute difference in variance in the data between groups (average over all measured genes).

**comparison**	**mean absolute logFC**	**mean absolute difference in variance between groups**
3M HET vs 3M WT	0.041	0.0042
9M HET vs 9M WT	0.029	0.0046
13M HET vs 13M WT	0.027	0.0037
9M HET vs 3M HET	0.048	0.0042
13M HET vs 9M HET	0.053	0.0044

Table S3: Genotype-dependent pathway alterations (HET vs WT), ranked in terms of their FDR. Fourth column: number of network objects from the data analyzed in this study.

age	pathway maps	total size	FDR	in DEGs
3M (top 10)	Neurophysiological process - HTR1A receptor signaling in neuronal cells[1]	43	1.816E-03	8
	Neurophysiological process - Activity-dependent synaptic AMPA receptor removal	64	1.037E-02	8
	Neurophysiological process - Constitutive and regulated NMDA receptor trafficking	65	1.037E-02	8
	Development - Epigenetic and transcriptional regulation of oligodendrocyte precursor cell differentiation and myelination	34	1.037E-02	6
	Regulation of lipid metabolism - Regulation of lipid metabolism via LXR, NF-Y and SREBP	38	1.528E-02	6
	PGE2 pathways in cancer[2]	55	1.528E-02	7
	Signal transduction - Angiotensin II/AGTR1 signaling via Notch, Beta-catenin and NF-kB pathways	76	1.683E-02	8
	Notch signaling in oligodendrocyte precursor cell differentiation in multiple sclerosis[3]	27	1.987E-02	5
	Development - Gastrin in cell growth and proliferation	62	2.306E-02	7
	G-protein signaling - Regulation of Cyclic AMP levels by ACM	45	2.379E-02	6
9M	no altered pathways			
13M	no altered pathways			

[1] HTR1A: synonym for 5-HT1A

[2] described as cancer pathway in GeneGO MetaCore^{TM}, but also an inflammation pathway in neurodegenerative diseases [3]

[3] described as pathway in multiple sclerosis in GeneGO MetaCore^{TM}, but also an important pathway in neurodegenerative diseases [1]

Table S4: Genotype-dependent GO biological process (BP) alterations (HET vs WT), ranked in terms of their FDR. Fourth column: number of network objects from the data analyzed in this study.

age	GO term	total size	FDR	in DEGs
3M (top 10)	GO:0050806: positive regulation of synaptic transmission	272	3.599E-09	31
	GO:0055067: monovalent inorganic cation homeostasis	198	1.536E-08	25
	GO:0030004: cellular monovalent inorganic cation homeostasis	159	2.710E-08	22
	GO:0006885: regulation of pH	140	7.045E-08	20
	GO:0007613: memory	240	3.622E-07	25
	GO:0030641: regulation of cellular pH	124	1.740E-06	17
	GO:0048167: regulation of synaptic plasticity	327	2.183E-06	28
	GO:0060251: regulation of glial cell proliferation	58	1.382E-05	11
	GO:0045927: positive regulation of growth	463	2.129E-05	32
	GO:0098739: import across plasma membrane	137	2.590E-05	16
9M (top 10)	GO:0090131: mesenchyme migration	12	1.272E-06	3
	GO:1903116: positive regulation of actin filament-based movement	16	1.527E-06	3
	GO:1904238: pericyte cell differentiation	17	1.527E-06	3
	GO:0061448: connective tissue development	343	3.948E-06	5
	GO:0002064: epithelial cell development	350	4.093E-06	5
	GO:0072132: mesenchyme morphogenesis	72	5.986E-05	3
	GO:1903115: regulation of actin filament-based movement	79	7.446E-05	3
	GO:0070374: positive regulation of ERK1 and ERK2 cascade	369	1.397E-04	4
	GO:0071300: cellular response to retinoic acid	134	2.680E-04	3
	GO:0070372: regulation of ERK1 and ERK2 cascade	481	3.312E-04	4
13M	no altered GO BP			

Table S5: Age-dependent pathway alterations, ranked in terms of their FDR. Fourth column: number of network objects from the data analyzed in this study. Type 1: DEGs increasing with age; type 2: DEGs decreasing with age; type 3a: DEGs with increased expression between 3M and 9M, and decreased expression between 9M and 13M; type 3b: DEGs with decreased expression between 3M and 9M, and increased expression between 9M and 13M.

type	pathway maps	total size	FDR	in DEGs
type 1	no pathway alterations			
type 2	no pathway alterations			
type 3a (top 10)	Development - Astrocyte differentiation from adult stem cells	40	1.987E-04	6
	Chemotaxis - Inhibitory action of lipoxins on IL-8- and Leukotriene B4-induced neutrophil migration	53	7.330E-04	6
	Nociception - Nociceptin receptor signaling	76	2.987E-03	6
	Development - TGF-beta-dependent induction of EMT via RhoA, PI3K and ILK	46	2.987E-03	5
	Development - S1P2 and S1P3 receptors in cell proliferation and differentiation	26	3.015E-03	4
	Chemotaxis - CCR1 signaling	53	3.241E-03	5
	Impaired inhibitory action of lipoxins on neutrophil migration in CF	56	3.912E-03	5
	Development - S1P1 receptor signaling via beta-arrestin	34	5.460E-03	4
	Stem cells - Response to hypoxia in glioblastoma stem cells	40	9.357E-03	4
	Stem cells - H3K27 demethylases in differentiation of stem cells	40	9.357E-03	4
type 3b	no pathway alterations			

Table S6: Age-dependent GO biological process (BP) alterations, ranked in terms of their FDR. Fourth column: number of network objects from the data analyzed in this study. Type 1: DEGs increasing with age; type 2: DEGs decreasing with age; type 3a: DEGs with increased expression between 3M and 9M, and decreased expression between 9M and 13M; type 3b: DEGs with decreased expression between 3M and 9M, and increased expression between 9M and 13M.

type	GO term	total size	FDR	in DEGs
type 1	no altered GO BP			
type 2	no altered GO BP			
type3a (top 10)	GO:0035588: G protein-coupled purinergic receptor signaling pathway	29	7.908E-11	9
	GO:0035587: purinergic receptor signaling pathway	38	6.608E-10	9
	GO:0001973: adenosine receptor signaling pathway	15	6.608E-10	7
	GO:0061448: connective tissue development	343	3.339E-09	18
	GO:0072132: mesenchyme morphogenesis	72	6.974E-09	10
	GO:0050768: negative regulation of neurogenesis	498	1.978E-08	20
	GO:0031279: regulation of cyclase activity	86	3.030E-08	10
	GO:0006171: cAMP biosynthetic process	17	7.385E-08	6
	GO:0045665: negative regulation of neuron differentiation	403	1.688E-07	17
	GO:0007188: adenylate cyclase-modulating G protein-coupled receptor signaling pathway	306	1.914E-07	15
type 3b (top 10)	GO:1990408: calcitonin gene-related peptide receptor signaling pathway	9	2.861E-05	3
	GO:0032730: positive regulation of interleukin-1 alpha production	10	3.063E-05	3
	GO:0097647: amylin receptor signaling pathway	13	4.629E-05	3
	GO:0097646: calcitonin family receptor signaling pathway	14	4.629E-05	3
	GO:0007218: neuropeptide signaling pathway	146	4.629E-05	5
	GO:0032650: regulation of interleukin-1 alpha production	15	4.629E-05	3
	GO:0045651: positive regulation of macrophage differentiation	23	1.493E-04	3
	GO:0002031: G protein-coupled receptor internalization	25	1.660E-04	3
	GO:0050961: detection of temperature stimulus involved in sensory perception	31	2.826E-04	3
	GO:0050965: detection of temperature stimulus involved in sensory perception of pain	31	2.826E-04	3

Table S7: Overlap DEGs 3M HET vs 3M WT with DEGs from a recent meta-analysis on PD in human [2]. Protein names were extracted from Uniprot database (https://www.uniprot.org/) [4]; the brain cell type where the genes show the highest expression was extracted from the Brain RNAseq database (https://www.brainrnaseq.org/) [5].

**gene symbol (human)**	**protein name (human)**	**cell type (human)**	**gene symbol (mouse)**	**protein name (mouse)**	**cell type (mouse)**
OLFM1	Noelin	neurons	Olfm1	Noelin	neurons
KIF3C	Kinesin-like protein KIF3C	neurons	Kif3c	Kinesin-like protein	neurons
NLK	Mitogen-activated protein kinase	neurons	Nlk	Serine/threonine-protein kinase NLK	endothelial
SLC25A16	Graves disease carrier protein	neurons	Slc25a16	Graves disease carrier protein homolog	astrocytes
SCAMP1	Secretory carrier-associated membrane protein 1	neurons	Scamp1	Secretory carrier-associated membrane protein	neurons
GNG3	Guanine nucleotide-binding protein G(I)/G(S)/G(O) subunit gamma-3	neurons	Gng3	Guanine nucleotide-binding protein G(I)/G(S)/G(O) subunit gamma-3	neurons
CXXC1	CXXC-type zinc finger protein 1	astrocytes	Cxxc1	Uncharacterized protein	endothelial
SNX10	Sorting nexin-10	neurons	Snx10	PX domain-containing protein	neurons
TUBA4A	Tubulin alpha chain	neurons	Tuba4a	Tubulin alpha-4A chain	microglia
STAU2	Double-stranded RNA-binding protein Staufen homolog 2	neurons	Stau2	Double-stranded RNA-binding protein Staufen homolog 2	neurons
LMO3	LIM domain only protein 3	astrocytes	Lmo3	LIM domain only protein 3	unknown
TUSC2	Tumor suppressor candidate 2	neurons	Tusc2	Tumor suppressor candidate 2	OPC
ATP6V1C1	V-type proton ATPase subunit C	neurons	Atp6v1c1	V-type proton ATPase subunit C	microglia
ARHGEF9	Rho guanine nucleotide exchange factor 9	neurons	Arhgef9	Rho guanine nucleotide exchange factor 9	neurons
SLC25A22	Mitochondrial glutamate carrier 1	astrocytes	Slc25a22	Mitochondrial glutamate carrier 1	neurons
PCDH8	Protocadherin-8	neurons	Pcdh8	Protocadherin-8	neurons
LIN7B	Protein lin-7 homolog B	endothelial	Lin7b	Protein lin-7 homolog B	neurons
SNAPC5	snRNA-activating protein complex subunit 5	astrocytes	Snapc5	snRNA-activating protein complex subunit 5	endothelial
SEH1L	Nucleoporin SEH1	neurons	Seh1l	Nucleoporin SEH1	neurons
ATXN7L3B	Ataxin-7-like protein 3B	neurons	Atxn7l3b	Ataxin-7-like protein 3B	pan-cellular
FBXO21	F-box only protein 21	neurons	Fbxo21	Uncharacterized protein	neurons
CDH19	Cadherin-19	oligodendrocytes	Cdh19	Uncharacterized protein	oligodendrocytes
PKNOX2	Homeobox protein PKNOX2	neurons	Pknox2	Meis_PKNOX_N domain-containing protein	neurons
GOT1	Aspartate aminotransferase, cytoplasmic	pan-cellular	Got1	Aspartate aminotransferase, cytoplasmic	pan-cellular
PID1	PTB-containing, cubilin and LRP1-interacting protein	neurons	Pid1	Uncharacterized protein	OPC
TOMM20	Mitochondrial import receptor subunit TOM20 homolog	neurons	Tomm20	Mitochondrial import receptor subunit TOM20 homolog	endothelial
CDC27	Cell division cycle protein 27	neurons	Cdc27	Uncharacterized protein	endothelial
ATP6V1E1	V-type proton ATPase subunit E 1	neurons	Atp6v1e1	V-type proton ATPase subunit E 1	unknown
PLD3	Phospholipase D3	astrocytes	Pld3	Phospholipase D3	microglia
ATP1A1	Sodium/potassium-transporting ATPase subunit alpha-1	neurons	Atp1a1	Sodium/potassium-transporting ATPase subunit alpha	endothelial
B4GALT6	Beta-1,4-galactosyltransferase 6	neurons	B4galt6	Uncharacterized protein	neurons
SCAMP5	Secretory carrier-associated membrane protein	neurons	Scamp5	Secretory carrier-associated membrane protein	oligodendrocytes
SMIM14	Small integral membrane protein 14	neurons	Smim14	Small integral membrane protein 14	oligodendrocytes
RNF10	RING finger protein 10	astrocytes	Rnf10	RING finger protein 10	OPC
MAPK1	Mitogen-activated protein kinase	neurons	Mapk1	Mapk1 protein	neurons
SLC35F1	Solute carrier family 35 member F1	neurons	Slc35f1	Solute carrier family 35 member F1	OPC
KANSL2	KAT8 regulatory NSL complex subunit 2	neurons	Kansl2	KAT8 regulatory NSL complex subunit 2	OPC
PELI1	E3 ubiquitin-protein ligase pellino homolog 1	microglia	Peli1	E3 ubiquitin-protein ligase pellino homolog	oligodendrocytes
CHD7	Chromodomain-helicase-DNA-binding protein 7	astrocytes	Chd7	Chromodomain-helicase-DNA-binding protein 7	OPC
MAN1C1	alpha-1,2-Mannosidase	neurons	Man1c1	alpha-1,2-Mannosidase	microglia
COPS8	COP9 signalosome complex subunit 8	neurons	Cops8	COP9 signalosome complex subunit 8	OPC
KDSR	3-ketodihydrosphingosine reductase	oligodendrocytes	Kdsr	Uncharacterized protein	oligodendrocytes
GMEB2	Alternative protein GMEB2	astrocytes	Gmeb2	Glucocorticoid modulatory element-binding protein 2	astrocytes
RPL38	60S ribosomal protein L38	oligodendrocytes	Rpl38	60S ribosomal protein L38	endothelial
SNRNP25	U11/U12 small nuclear ribonucleoprotein 25 kDa protein	neurons	Snrnp25	U11/U12 small nuclear ribonucleoprotein 25 kDa protein	oligodendrocytes

References

[1] Ettle, B., Schlachetzki, J.C., Winkler, J.: Oligodendroglia and myelin in neurodegenerative diseases: more than just bystanders? Molecular neurobiology **53**(5), 3046–3062 (2016)

[2] Kelly, J., Moyeed, R., Carroll, C., Albani, D., Li, X.: Gene expression meta-analysis of Parkinson's disease and its relationship with Alzheimer's disease. Molecular brain **12**(1), 16 (2019)

[3] Lima, I.V.d.A., Bastos, L.F.S., Limborço-Filho, M., Fiebich, B.L., de Oliveira, A.C.P.: Role of prostaglandins in neuroinflammatory and neurodegenerative diseases. Mediators of inflammation **2012**, 946813 (2012)

[4] UniProt Consortium, et al.: UniProt: the universal protein knowledgebase. Nucleic acids research **46**(5), 2699 (2018)

[5] Zhang, Y., Chen, K., Sloan, S.A., Bennett, M.L., Scholze, A.R., O'Keeffe, S., Phatnani, H.P., Guarnieri, P., Caneda, C., Ruderisch, N., et al.: An RNA-sequencing transcriptome and splicing database of glia, neurons, and vascular cells of the cerebral cortex. Journal of Neuroscience **34**(36), 11929–11947 (2014)
